# Examining indicators of psychosocial risk and resilience in parents of autistic children

**DOI:** 10.3389/fnbeh.2023.1102516

**Published:** 2023-05-15

**Authors:** Amanda Dimachkie Nunnally, Reina S. Factor, Alexandra Sturm, Latha Valluripalli Soorya, Allison Wainer, Sandra Taylor, Matthew Ponzini, Leonard Abbeduto, Amanda C. Gulsrud

**Affiliations:** ^1^Department of Psychiatry and Behavioral Sciences, MIND Institute, University of California, Davis, Sacramento, CA, United States; ^2^Semel Institute for Neuroscience and Human Behavior, University of California, Los Angeles, Los Angeles, CA, United States; ^3^Department of Psychological Science, Loyola Marymount University, Los Angeles, CA, United States; ^4^Department of Psychiatry and Behavioral Sciences, Rush University Medical Center, Chicago, IL, United States; ^5^Department of Public Health Sciences, School of Medicine, University of California, Davis, Davis, CA, United States

**Keywords:** autism spectrum disorder, parenting, risk, resilience, emotion regulation

## Abstract

**Background:**

Parents of autistic children experience increased levels of caregiver strain and adverse mental health outcomes, even in comparison to parents of children with other neurodevelopmental disabilities. Previous studies have largely attributed these increased levels of mental health concerns to their child behavioral concerns and autism symptomatology, but less attention has been given to other potential child factors, such as child adaptive functioning. Additionally, little is known about potential protective factors, such as parents’ emotion regulation (ER) abilities, that may ameliorate the experience of caregiver strain, anxiety, and depression.

**Objective:**

The current study examined the impact of child characteristics (restricted and repetitive behaviors, adaptive functioning and behavioral concerns) on parent mental health outcomes (caregiver strain, anxiety, depression and wellbeing). Additionally, we explore parents’ ER abilities as a moderator of the impact of child characteristic on parents’ mental health outcomes.

**Results:**

Results of linear mixed effect models indicated a significant relationship between parents’ ER abilities and all four parent outcomes. Additionally, children’s adaptive functioning abilities and repetitive behaviors (RRBs) were significant predictors of caregiving strain. Parents’ ER abilities were a significant moderator of the effect of children’s repetitive behaviors and adaptive functioning challenges on caregiver strain, such that better ER abilities mitigated the impact of child clinical factors on caregiver strain. Finally, a significant difference was detected for mothers’ and fathers’ mental health, with mothers reporting higher caregiver strain, and more symptoms of anxiety and depression than did fathers.

**Conclusion:**

This study leveraged a large sample of autistic children and their biological parents to examine the relationship between children’s clinical characteristics and parents’ psychological wellbeing. Results indicate that, although parents of autistic children do experience high rates of internalizing mental health concerns that relate to child adaptive functioning and RRBs, parent ER abilities act as a protective factor against parents’ adverse mental health outcomes. Further, mothers in our sample reported significantly higher rates of depression, anxiety, and caregiver strain, as compared with fathers.

## Introduction

Parents of autistic^1^ children experience increased levels of caregiver strain and higher rates of general mental health concerns compared to parents of neurotypical (NT) children, and those with other neurodevelopmental disabilities (Abbeduto et al., 2004; Davis and Carter., 2008; Estes et al., 2009, 2013; Hartley et al., 2012). Several studies have reported increased rates of depression (Abbeduto et al., 2004; Hartley et al., 2012; Cohrs and Leslie, 2017) among parents of autistic children. Additionally, in one study of 52 parents of autistic children, researchers reported that 53.8% of the parents show clinically significant mental health concerns (Nikmat et al., 2008), with mothers reporting a greater adverse impact on psychological wellbeing compared to fathers (Nikmat et al., 2008). Understanding parents’ caregiver strain, mental health, and wellbeing is particularly important, as research has demonstrated that parents play an important role in scaffolding their children’s development (e.g., maternal sensitivity influences child expressive language growth; Greenberg et al., 2006; Baker et al., 2010). Caregiver strain is also associated with greater use of maladaptive parenting styles that may exacerbate behavioral regulation difficulties in autistic children (Hutchison et al., 2016). Thus, ignoring parental mental health needs will make it difficult to support child development because of the transactional nature of the relationships among the members of a family system (Gulsrud et al., 2010).

Most of the prior literature exploring predictors of parents’ caregiver strain, mental health, and wellbeing have focused primarily on the impact of autistic children’s autism symptom severity. Specifically, higher rates of adverse parental mental health and increased caregiver strain have previously been attributed to perceived behavioral challenges associated with their child’s autism (Smith et al., 2008; Lyons et al., 2010; Ingersoll and Hambrick, 2011; Hartley et al., 2012; He et al., 2022; Porter et al., 2022). However, less attention has been given to other parent and child factors that may impact caregiver strain and mental health in parents (e.g., wellbeing, depression, anxiety). Further, few studies have explored how parent factors may mitigate the adverse impact of other child factors on mental health and wellbeing in parents of autistic children (Weiss et al., 2012). The present study explored the impact of child and parent-specific factors on parents’ caregiver strain, mental health, and wellbeing. Additionally, we examine the potential mitigating role of parents’ emotion regulation on the impact of child factors on parents’ mental health and wellbeing.

### Predictors of caregiver strain, mental health, and wellbeing

Factors related to increased caregiver strain among parents of autistic children have been explored in numerous reports, with children’s cognitive challenges and severity of autism symptoms being associated with increased caregiver strain (Abbeduto et al., 2004; Lecavalier et al., 2006; Davis and Carter., 2008; Rao and Beidel, 2009; Hartley et al., 2012; Karst and Van Hecke, 2012; Smith et al., 2012) and depression (Benson and Karlof, 2009; Ingersoll and Hambrick, 2011). Additionally, children’s behavioral concerns, such as dysregulation and externalizing behaviors, have been found to be highly correlated with caregiver strain in parents of autistic children (Davis and Carter., 2008), which in turn was associated with parents’ higher levels of anxiety and depression (Rezendes and Scarpa, 2011). Increased caregiver strain is also related to increased rates of children’s restricted and repetitive behaviors (RRBs), with parents citing difficulty managing these behaviors adding to their own caregiver strain (Mercier et al., 2000; Gabriels et al., 2005; Lecavalier et al., 2006; Bishop et al., 2007). Furthermore, studies suggest that greater parental involvement in a child’s day-to-day activities (e.g., involvement with daily routine, school, domestic duties) may lead to greater caregiver strain and negatively impact the family system (Tehee et al., 2009).

### Resilience of parents of autistic children

Data also suggest resiliency, or the ability to positively adapt to the environment in the face of adversity or challenge (Luthar et al., 2000), in many parents of autistic children. Specifically, positive meaning-making (e.g., diagnosis as a strength for the family unit, acknowledging available resources) of the experience as caregivers of autistic children can buffer against stressful situations (Barakat and Linney, 1992; Anuradha, 2004; Wilgosh and Scorgie, 2006). Further, research has demonstrated that social support, hope and spirituality and religiosity are important protective factors for caregiver strain, mental health and wellbeing (Lyons et al., 2010; Ekas et al., 2016; Slattery et al., 2017; Pepperell et al., 2018). Although research has identified a range of resilience factors for parents of autistic children, little is known regarding the impact of parent emotion regulation abilities on caregiver strain and wellbeing in this population.

### Emotion regulation as resilience

Emotion regulation (ER) is an umbrella term that refers to the ability to monitor, evaluate and modify emotional reactions (Gross, 1998). In the general population of NT adults, difficulties with ER have been associated with a range of mental health concerns, including increased rates of anxiety and depression (Mennin et al., 2007; Etkin et al., 2010; Cludius et al., 2020). Additionally, ER abilities have been associated with indices of wellbeing in NT adults, with lower ER abilities predicting lower self-reported wellbeing (Haga et al., 2009; Mandal et al., 2011).

Despite the research supporting the impact of ER abilities on individuals’ mental health and wellbeing in the general population, few studies have examined these associations in parents. These studies find that ER abilities play an important role in supporting parents’ mental health and wellbeing. In one recent study of caregivers of NT children during the COVID-19 pandemic, caregivers’ ER abilities predicted their mental health 2 months later (Russell et al., 2022). In another study, researchers provided an online ER intervention to parents, targeting the use of adaptive ER strategies (Preuss et al., 2021). Findings revealed that parents who received the intervention had a significant decrease in parenting stress at follow-up in comparison to the wait-list control group (Preuss et al., 2021). With regard to parents of autistic children, even less is known. To our knowledge, one study to date has examined the association between ER and parenting stress in parents of autistic children. In this study, Hu et al. (2019) found a significant, negative association between parents’ ER abilities and their self-reported parenting stress. Further research is needed to better understand the impact of ER abilities on mental health and wellbeing for parents of autistic children.

### Mothers and fathers of autistic children

In studies of the general population of heterosexual couples, fathers play important, but distinct, and complementary roles to mothers in many families. However, fathers of children with developmental disabilities, including autism, have been studied infrequently regarding their roles and relationships within the family. In the studies that have been conducted, child characteristics, partner characteristics, and features of the marital relationship have been found to differentially affect mothers and fathers of children with autism or other disabilities (Bristol et al., 1988; Hartley et al., 2016). There is evidence, for example, that fathers are more negatively affected by their autistic child’s behavioral concerns than are mothers (Davis and Carter., 2008). Moreover, it is likely that the psychological state of one parent may affect that of the other parent in two-parent families (Hastings, 2003). In fact, maternal symptoms of depression have been found to predict paternal psychological wellbeing in families with autistic children (Hartley et al., 2012). Research suggests that mothers and fathers also differ in their adaptability (Bendixen et al., 2011) and in the types of support they provide for their families (emotional vs. practical support, respectively; Seligman and Darling, 2009).

There are inconsistent findings with respect to differences in caregiver strain, mental health, and wellbeing between fathers and mothers of autistic children. Studies examining mental health outcomes in parents of autistic children have largely found that mothers report higher levels of depression (Hastings et al., 2005; Ozturk et al., 2014; Foody et al., 2015; Cohrs and Leslie, 2017; Li et al., 2022) and anxiety (Hastings, 2003; Foody et al., 2015; Li et al., 2022) than do fathers of autistic children. Less consistent findings have been found regarding caregiver strain or stress, with some studies reporting higher rates of parenting stress in mothers as compared with fathers (Moes et al., 1992; Sharpley et al., 1997; Tehee et al., 2009; Dabrowska and Pisula, 2010; Falk et al., 2014). Other studies have reported no significant difference in parenting stress between mothers and fathers of autistic children (Hastings, 2003; Davis and Carter., 2008; Nikmat et al., 2008; Ozturk et al., 2014). These studies typically involve small samples thus, they have had limited statistical power to detect differences between mothers and fathers and are limited in potential generalizability. Moreover, studies involving fathers have not focused on the role of ER, or the potential protective factors associated with positive outcomes. Overall, the inconsistencies in these data demonstrate a need for further research into the unique role of mothers and fathers of autistic children.

### The current study

This study was designed to leverage a large sample to explore the relationship between autistic children’s behavioral characteristics and parents’ psychosocial risk and resilience, as well as to evaluate the potential role of ER as a protective factor for parents. In addition, mothers and fathers from the same family are included in analyses, thereby allowing exploration of differences in their psychosocial risk and resilience. It was hypothesized that children’s higher RRBs and behavioral concerns, and lower adaptive functioning abilities (i.e., tasks of daily living), would be associated with increased caregiver strain and adverse mental health. Additionally, it was hypothesized that higher ER abilities in parents would be associated with lower levels of caregiver strain and more positive mental health outcomes, and that ER would moderate the association between child factors and indicators of parents’ mental health and wellbeing. Lastly, it was hypothesized that mothers in our sample would report significantly higher rates of caregiver strain, anxiety and depression than would fathers.

## Materials and methods

### SPARK cohort

Beginning in April 2016, SPARK began a nationwide recruitment effort with 21 clinical sites (growing to include 31 in 2021) and an extensive social media campaign. Any individual living in the United States with a professional diagnosis of ASD (obtained from a provider or through school), along with their parents and an unaffected sibling, are eligible to participate in SPARK. Phenotypic data and biospecimens are collected remotely, with online access to the study protocol, making participation more accessible and convenient. Participants consent to share their de-identified data, and to be contacted for future ASD-related research studies for which they may be eligible. Additionally, participants may consent to contribute a saliva sample for genetic analysis and may opt to receive individual genetic results related to ASD, in the event that a primary genetic cause of ASD is identified. For a detailed description of genetic material collection, genomic analyses and return of results to participants see Feliciano et al.’s (2019) publication. SPARK participants are also asked to complete a battery of online questionnaires.

### SPARK research match

The data for the current project was obtained through the SPARK Research Match program, which connects qualified members of the SPARK community with research studies, inviting them to volunteer as participants. Data were accessed by submitting an application to SFARI Base, describing the aims of the study, as well as a description of the inclusion criteria for the proposed project. The application was reviewed and approved by the SPARK Participant Access Committee (PAC), and participants who met study criteria were invited to participate in the current study. Data collection took place between January and February of 2021, during which time the participants completed a battery of online surveys regarding proband characteristics, in addition to psychological risk and resilience for both biological parents.

### Participants

The overall SPARK sample included 106,577 probands, the parents of whom were invited to participate in the current study if they met the following criteria: (1) the proband was less than 18 years old and (2) both biological parents were available to participate. This resulted in a sample of 263 dyads (biological mother, biological father) that participated in the online survey study. Probands were largely male (77.9%) and had an average age of 7.37 years (*SD* = 3.92; range = 1–17 years). A majority of probands identified as White (63.9%), with the remainder identifying as Asian (1.1%), African American (4.2%) and Other (3.4%). A portion of the sample identified as having multiple races (9.5%), and 17.9% did not endorse a race. Lastly, 11.0% of the sample identified as ethnically Hispanic. Maternal and paternal demographics are presented in Table 1.

**TABLE 1:** Mother and father demographic information.

	Mothers		Fathers	
	**Mean**	**SD**	**Mean**	**SD**
CA (Years)	40.64	7.014	42.88	7.743
	Frequency	%	Frequency	%
**Marital status**				
Married	242	92%	239	90.9%
Widowed	1	0.4%	0	0%
Divorced	5	1.9%	10	3.8%
Separated	2	0.8%	2	0.8%
Never married	3	1.1%	3	1.1%
Living with a partner	10	3.8%	9	3.4%
**Education**				
Did not attend high school	1	0.4%	0	0%
Some high school	1	0.4%	14	5.3%
High school graduate	26	9.9%	39	14.8%
GED diploma	8	3.0%	13	4.9%
Trade school	16	6.1%	14	5.3%
Some college	45	17.1%	42	16%
Associate degree	22	8.4%	18	6.8%
Baccalaureate degree	76	28.9%	56	21.3%
Graduate or professional degree	68	25.9%	67	25.5%
**Occupation**				
Not currently employed/Unable to work	26	9.9%	19	7.2%
Full time care provider	109	41.4%	10	3.8%
Part-time/Temporary worker	27	10.3%	5	1.9%
Laborer	4	1.5%	35	13.3%
Service industry	5	1.9%	14	5.3%
Craftsmen/Skilled technician/Clerical/Office worker	23	8.7%	47	17.9%
Supervisor/Middle management	22	8.4%	39	14.8%
Upper management/administration/Professional	43	16.3%	79	30%
Executive	4	1.5%	14	5.3%

### Child measures

Child Behavior Checklist (CBCL; Achenbach and Rescorla, 2001). The Child Behavior Checklist, now known as the *Achenbach System of Empirically Based Assessments*, is a parent report questionnaire that measures the presence of behavioral and emotional challenges in children. The most recent version of the CBCL includes two separate forms, one to be used with children between the ages of 1.5 and 5 years and the second to be used with children between the ages of 6 and 18. Both forms of the CBCL have been found to have strong internal consistency (α = 0.92–0.94) and test-retest reliability in the norming sample (*r* = 0.89–0.92; Achenbach and Rescorla, 2001). Additionally, both scales have been demonstrated to have acceptable construct and criterion validity (Achenbach and Rescorla, 2001). The assessment yields scores across 6 different scales (affective problems, attention-deficit/hyperactivity, anxiety, oppositional defiance, somatic problems, and conduct problems), and three composite scores (Internalizing problems, Externalizing problems and Total Problems). For the purposes of the current study, the Total Problems score was used as a measure of children’s emotional and behavioral concerns (hereafter referred to as behavioral concerns for readability). The choice was made to exclude subscales from the analyses, as two different forms of the CBCL were used (1.5–5 and 6–18) which include different subscales.

Repetitive Behavior Scale–Revised (RBS-R; Bodfish et al., 1999). The RBS-R is an informant-report instrument that measures the presence and severity of restricted and repetitive behaviors, consisting of 43 items across six subscales (Stereotyped Behavior, Self-injurious behavior, Compulsive Behavior, Routine Behavior, Sameness Behavior, and Restricted Behavior). This measure was completed by parents, who rated their child’s behaviors on a 4-point Likert scale, ranging from “0” (behavior does not occur) to “3” (behavior is a severe problem). The measure was found to have good inter-rater reliability (*r* = 0.88) and test-retest reliability in the norming sample (*r* = 0.71; Bodfish et al., 1999). The RBS-R yields an overall total raw score, based on summed items scores across subscales, which was used in this study as a measure of individual’s restricted, repetitive behaviors.

Vineland Adaptive Behavior Scale–Third Edition (VABS-3; Sparrow et al., 2016). The VABS-3 is considered the gold-standard measure of adaptive functioning for individuals from birth to 90 years old. Parents in the current study completed the caregiver questionnaire version of the assessment, which has been found to have strong internal consistency (α = 0.96–0.99) and acceptable test-retest reliability in the norming sample (*r* = 0.80–0.93; Sparrow et al., 2016). The measure has been shown to correlate with other measures of adaptive functioning (Sparrow et al., 2016), including the Bayley Scales of Infant and Toddler Development (Bayley-III; Bayley, 2006) and the Adaptive Behavior Assessment System (ABAS-3; Harrison and Oakland, 2015). The VABS-3 provides composite standard scores (*M* = 100, *SD* = 15) across four domains (Communication, Daily Living Skills, Socialization and Motor), in addition to an overall Adaptive Behavior Composite (ABC). The ABC standard score was used for the current study as a measure of overall adaptive functioning. We did not include analyses of the subdomains of the VABS-3 because 3 of the 4 subdomains are not normed for children under the age of 3 years, which would exclude part of our sample.

### Parent measures

Caregiver Strain Questionnaire–Short Form (CGSQ-SF; Brannan et al., 1997). The CGSQ-SF is a self-report measure that assesses parenting strain in the previous month. It consists of 10 items derived from the original long form, measuring strain across two subscales: Objective strain (6 items) and Subjective Internalized strain (4 items). Response options are in the form of a 5-point Likert scale, ranging from *“Not a problem”* (1) to *“Very much a problem”* (5). The CGSQ-SF Total Score, a score derived from the mean of all items, was used in this study to characterize overall caregiving strain. The CGSQ-SF has strong psychometric properties, with an internal consistency reliability coefficient of 0.90 (Brannan et al., 2012).

Patient Health Questionnaire (PHQ-9; Kroenke and Spitzer, 2002). The PHQ-9 is a self-report questionnaire measuring depression, adapted from the depression module of the PRIME-MD diagnostic instrument for common mental disorders. It consists of 9 items, each of which represents one of nine DSM-IV criteria for major depression. Item responses are measured using a 3-point Likert scale, ranging from *“Not at all”* (1) to *“Nearly every day”* (3). The PHQ-9 was found to have strong internal reliability (0.86–0.89; Kroenke and Spitzer, 2002) and test-retest reliability in the norming sample (0.84; Kroenke and Spitzer, 2002). It was also found to have strong construct and criterion validity (Kroenke and Spitzer, 2002). The measure yields a total score that represents the severity of respondents’ depression symptoms.

Generalized Anxiety Disorder (GAD-7; Spitzer et al., 2006). The GAD-7 is a self-report questionnaire that assess symptoms of generalized anxiety, asking participants how often in the previous 2 weeks they have been bothered by anxiety symptoms. The measure consists of 7 items, scored on a 4-point Likert scale ranging from *“Not at all”* (0) to *“Nearly every day”* (3). The measure was found to have strong psychometric properties, with good internal consistency (Cronbach α = 0.92; Spitzer et al., 2006) and test-retest reliability in the norming sample (ICC = 0.83; Spitzer et al., 2006). Additionally, this measure was found to have strong evidence of criterion, construct and factorial validity (Spitzer et al., 2006). The measure yields a total score between 0 and 21, with higher scores indicated greater anxiety symptomatology.

Wellbeing Scale (WBS; Ryff and Keyes, 1995; Keyes et al., 2002). A modified version of Ryff’s Scales of Psychological Well Being, the Wellbeing Scale includes a total of 18 items across 6 aspects of wellbeing: self-acceptance, autonomy, environmental mastery, purpose in life, positive relations with others, and personal growth. Responses are measured using a 7-point Likert scale ranging from *“Strongly agree”* (1) to *“Strongly disagree”* (7). While there have been mixed findings on the psychometrics of the modified form (Ryff and Keyes, 1995; Springer and Hauser, 2006), it has been widely used in the literature examining wellbeing in diverse samples (Clarke et al., 2001; Sagone and De Caroli, 2014; Khanjani et al., 2018). The measure yields a total mean score, such that a higher score reflects greater wellbeing.

Barkley Deficits in Executive Functioning Scale (BDEFS; Barkley, 2011b). The BDEFS is a self-report measure of individuals’ executive functioning abilities. It is comprised of 89 items that are answered using a 4-point Likert scale ranging from “1” (rarely or not at all) to “4” (very often). It consists of items across five subscales (self-management of time, self-organization/problem-solving, self-restraint, self-motivation and self-regulation of emotions). The measure is reported to have excellent internal consistency in the norming sample (α = 0.92; Barkley, 2011b). For the purposes of the current study, we present scores from the self-regulation of emotions subscale (13 items), as a measure of participants’ ER. Included in the self-regulation of emotions subscale are items designed to measure participants’ ability to regulate negative emotions, such as “Have trouble calming myself down once I am emotionally upset” and “Unable to manage my emotions in order to accomplish my goals successfully or get along with others.” Scores are calculated as a sum of scores across items, such that higher scores indicate poorer ER.

### Data analysis

Preliminary analyses were conducted to provide sample demographics and summary statistics (means and standard deviations) of all variables of interest. Data were analyzed to confirm that all assumptions of linear mixed effects models were met.

We used linear mixed effects models to assess the contribution of child characteristics to caregiving strain and mental health. Each parental outcome (CGSQ-SF, PHQ, GAD, and WBS) was modeled separately. We first modeled each parental outcome (CGSQ-SF, PHQ, GAD, and WBS) as a function of child characteristics (CBCL, VABS, and RBS-R), parent (mother/father) and all two-way interactions between child characteristics and parent. A random effect was included for each child to account for within-child correlation. Because four outcomes were analyzed, a Bonferroni corrected alpha level of 0.0125 was used to determine significance. If no interactions were significant, main effect only models were fit; otherwise, all interactions were retained in the model. We then tested the possible moderating effect of parents’ ER abilities by including BDEFS-ER as a main effect and two-way (BDEFS-ER*Child characteristic) and three-way interactions (BDEFS-ER*Parent*Child characteristic). All predictors and the moderator were centered and scaled to a mean of 0 and standard deviation of 1. Moderation models were also evaluated at a Bonferroni significance level of 0.0125. Main effects only models were fit if no interactions were significant, but all interactions retained if some were significant. Analyses were conducted using R Statistical Software version 4.2.0.

## Results

### Child and parent characteristics

On average, children in this sample had a mean Adaptive Behavior Composite Standard Score (ABC-SS) that fell in the “moderately low” range (*M* = 71.53, *SD* = 15.18), and a mean Total Problems score in the “clinical” range (*M* = 65.63, *SD* = 8.94). Additionally, children in the sample had a mean score of 33.85 on the RBS-R (out of a possible score of 129), with a large amount of variability (*SD* = 19.66).

Parents in this sample reported symptoms of mild depression and mild anxiety, as measured by the PHQ-9 and GAD-7, respectively. Additionally, both mothers and fathers in this sample had a CGSQ total score that fell in the “medium” range (see Table 2).

**TABLE 2:** Mother and father indicators of risk and resilience.

	Mothers		Fathers	
	**Mean**	**SD**	**Mean**	**SD**
CSQ-SF total score	2.81	0.977	2.63	0.920
PHQ-9–Total score	7.64	5.855	6.73	5.998
GAD-7–Total score	8.08	5.701	6.61	5.227
BDEF-SF–ER	22.98	8.305	23.32	7.996
Wellbeing scale–Total	5.10	0.837	5.015	0.880

### Associations between parental outcomes and child characteristics

**FIGURE 1:**
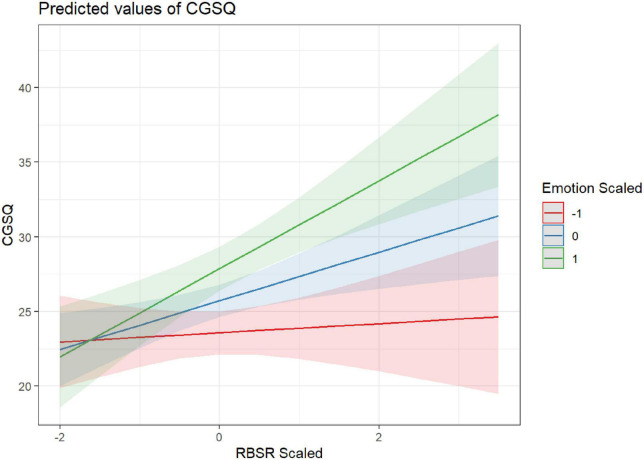
ER moderating the effect of RRBs on CGSQ.

**FIGURE 2:**
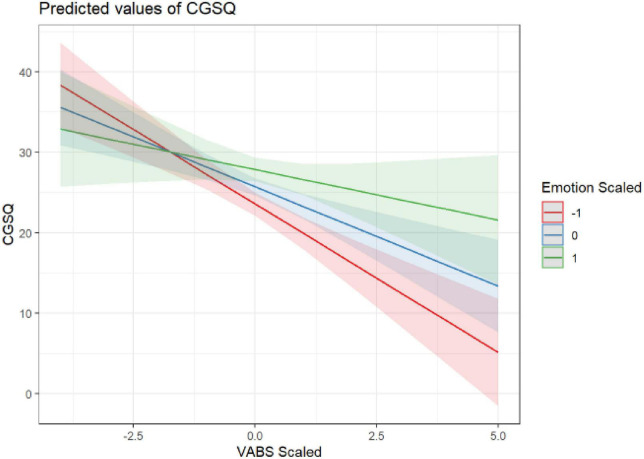
ER moderating the effect of VABS on CGSQ.

Two child characteristics, adaptive functioning (VABS-3) and repetitive behaviors (RBS-R), significantly predicted parents’ caregiver strain (CGSQ-SF), such that higher caregiver strain was associated with children’s lower adaptive functioning (β = −2.57; *p* < 0.001) and higher repetitive behaviors [(β = 2.12; *p* < 0.001; see Table 3)]. These relationships were not found to differ significantly by parent (Supplementary Table 1). Additionally, parents’ ER abilities moderated the effect of children’s RRBs on caregiving strain (β = 1.32; *p* = 0.002), such that parents with higher ER abilities (indicated by lower scored on BDEFS-ER) experienced smaller increases in caregiver strain for a given increase in a child’s RRBs. Although not statistically significant at the Bonferroni corrected level of 0.0125, there was some indication that the moderating effect of BDEFS-ER was considerably less for mothers than fathers (β = 1.49; *p* = 0.016). Similarly, parents’ ER abilities also moderated the effect of children’s adaptive functioning abilities on parents’ caregiver strain (β = 1.22; *p* = 0.011), such that parents with higher ER abilities experienced lower levels of caregiver strain in response to their children’s lower adaptive functioning abilities. See Figures 1, 2 and Table 4 for more information.

**TABLE 3:** Results of main effects only linear mixed effect models evaluating the effect of child characteristics on the four parent outcomes.

	CGSQ-SF			GAD-7			PHQ-9			WBS		
	**Estimates**	**CI**	***P***	**Estimates**	**CI**	***P***	**Estimates**	**CI**	***P***	**Estimates**	**CI**	***P***
Intercept	26.09***	24.98–27.19	<0.001	6.66***	5.97–7.34	<0.001	6.78***	6.04–7.53	<0.001	5.02***	4.91–5.13	<0.001
VABS	−2.57***	−3.54 –1.60	<0.001	0.01	−0.52–0.54	0.968	0.15	−0.45–0.75	0.627	0.02	−0.07– 0.10	0.722
CBCL	−0.68	−1.63–0.26	0.154	0.02	−0.49–0.54	0.932	−0.11	−0.70–0.47	0.701	−0.04	−0.13–0.04	0.328
RBS-R	2.12***	1.15–3.10	<0.001	0.92**	0.39–1.44	0.001	0.97**	0.37–1.57	0.002	−0.13*	−0.21–0.04	0.005
Parent (Mother)	2.06***	0.92–3.20	<0.001	1.66**	0.76–2.56	<0.001	1.01	0.08–1.93	0.033	0.05	−0.08–0.18	0.456

Bonferroni-corrected significance levels were used to control the Type I error rate across the four outcome models.

*p < 0.0125; **p < 0.0025; ***p < 0.00025.

**TABLE 4:** Results of mixed effect models evaluating the moderating effect of parents’ ER on the association between child characteristics and caregiver strain.

	CGSQ-SF		
	**Estimates**	**CI**	***P***
Intercept	25.75***	24.67–26.82	<0.001
VABS	−2.47***	−3.61–1.33	<0.001
CBCL	−1.03	−2.10–0.04	0.059
RBS-R	1.63*	0.52–2.73	0.004
Parent (Mother)	2.44***	1.36–3.51	<0.001
ER	2.16***	1.18–3.14	<0.001
VABS*Parent	−0.28	−1.41–0.85	0.631
VABS*ER	1.22*	0.27–2.16	0.011
CBCL*Parent	0.72	−0.33–1.78	0.178
CBCL*ER	−0.92	−1.87–0.02	0.055
RBS-R*Parent	0.28	−0.82–1.39	0.613
RBS-R*ER	1.32**	0.47–2.17	0.002
Parent*Emotion	−0.09	−1.44–1.25	0.891
VABS*Parent*ER	−0.38	−1.72–0.96	0.582
CBCL*Parent*ER	1.26	0.06–2.47	0.040
RBS-R*Parent*ER	−1.49	−2.70–0.28	0.016

Bonferroni-corrected significance levels were used to control the Type I error rate across the four outcome models.

*p < 0.0125; **p < 0.0025; ***p < 0.00025.

For the models of parents’ depression, anxiety and wellbeing, RBS-R was a significant predictor (Table 3), with increases in RRBs associated with higher anxiety (β = 0.92; *p* = 0.001) and depression (β = 0.97; *p* = 0.002), and lower wellbeing (β = −0.13; *p* = 0.005), although these results did not remain significant upon the inclusion of BDEFS-ER in the models. Additionally, these relationships did not differ significantly between mothers and fathers (Supplementary Table 1).

Parents’ ER abilities significantly predicted parents’ depression (β = 3.23; *p* < 0.001) and anxiety (β = 3.27; *p* < 0.001), such that parents with higher ER abilities had fewer symptoms of depression and anxiety. Additionally, a significant effect for parents’ ER was seen for parents’ wellbeing (β = −0.13; *p* = 0.005), with higher ER predicting higher wellbeing. See Table 5 for details. There was no evidence of different effects of ER on depression, anxiety and wellbeing between mothers and fathers (Supplementary Table 2).

**TABLE 5:** Results of main effects only linear mixed effect models evaluating the effect of parents’ ER and child characteristics on parent mental health outcomes.

	GAD-7			PHQ-9			WBS		
	**Estimates**	**CI**	***P***	**Estimates**	**CI**	***P***	**Estimates**	**CI**	***P***
Intercept	6.61***	6.06–7.17	<0.001	6.74***	6.12–7.36	<0.001	5.02***	4.93–5.11	<0.001
VABS	−0.27	−0.70–0.16	0.213	−0.14	−0.64–0.36	0.574	0.05	−0.02–0.12	0.189
CBCL	0.12	−0.30–0.53	0.577	−0.05	−0.53–0.44	0.852	−0.05	−0.12–0.02	0.184
RBS-R	0.44	0.01–0.87	0.043	0.47	−0.03–0.97	0.064	−0.06	−0.13–0.01	0.114
Parent (Mother)	1.77***	1.03–2.50	<0.001	1.07**	0.29–1.84	0.007	0.05	−0.07–0.17	0.429
ER	3.23***	2.84–3.63	<0.001	3.27***	2.84–3.71	<0.001	−0.43***	−0.50 –0.37	0.001

Bonferroni-corrected significance levels were used to control the Type I error rate across the four outcome models.

*p < 0.0125; **p < 0.0025; ***p < 0.00025.

Lastly, a significant main parent effect was detected, such that mothers reported experiencing higher caregiver strain (β = 2.06; *p* < 0.001) and anxiety (β = 1.66; *p* < 0.001) than fathers. See Table 3 for further details on all models.

## Discussion

The current study examined contributors to, and potential mitigators of, strain, wellbeing, and mental health outcomes in parents of autistic children. Consistent with previous findings in the literature, parent self-reported caregiving strain was associated with children’s adaptive functioning abilities and restricted and repetitive behaviors (RRB) (Bishop et al., 2007). In previous studies, increased rates of RRBs have been associated with higher levels of caregiving strain and difficulty managing RRBs in parents of autistic children (Gabriels et al., 2005; Lecavalier et al., 2006; Bishop et al., 2007; Nikmat et al., 2008). Similarly, challenges with adaptive functioning skills can greatly impact a child’s level of independence (Harrop et al., 2016) which, in turn, places greater caregiving burden on parents. One study reported that increased caregiving strain was associated with lower levels of child adaptive functioning, specifically in the domain of daily living skills (Postorino et al., 2019). While the current study did not examine the individual subscale scores for the RBS-R and VABS-3 due to methodological concerns (see methods for details), future research is needed to better understand how specific aspects of RRBs and adaptive functioning abilities uniquely contribute to parents’ mental health and wellbeing. Altogether, our findings are consistent with the extant literature regarding the relationship between children’s RRBs, adaptive functioning abilities and caregiving strain.

The current study did not find a significant association between children’s behaviors and caregiving strain. Initially, this lack of association appears at odds with previous studies that have shown that children’s behaviors and autism symptomatology are associated with caregiving strain in parents of autistic children (Davis and Carter., 2008; Lecavalier et al., 2006). However, the current study differs from previous studies by analyzing children’s RRBs alongside children’s behaviors as a predictor of caregiving strain. It is possible that the inclusion of children’s RRBs accounted for variance accounted for by children’s behaviors in previous studies. Therefore, there is a need to better understand the ways different categories of behaviors may influence caregiver strain and, specifically, how autistic children’s RRBs may be perceived as behavioral concerns by caregivers. The use of parent-report measures of child factors in this study may have also contributed to differences in associations between children’s behaviors and parents’ caregiver strain. Future studies would benefit from the inclusion of observational measures of children’s behaviors, alongside caregiver report measures, to offer multiple vantages of children’s behaviors. Such an approach would allow for a more detailed understanding of the specific behaviors which appear to impact caregiver strain. Additionally, physiological measures of stress (such as heart rate variability), have been found to provide a more objective method for studying individual differences and underlying biological influences in stress reactivity and ER (Factor et al., 2017). This would inform possible supports to help families address these challenges.

In the current study, child factors were significantly associated with parents’ caregiver strain, even after the inclusion of parents’ ER in the model. This finding is consistent with previous studies in which child-related factors have been consistently associated with caregiver strain in parents of autistic children (Gabriels et al., 2005; Lecavalier et al., 2006; Bishop et al., 2007; Falk et al., 2014). Child factors, specifically children’s RRBs, were also associated with parents’ anxiety, depression and wellbeing, but these findings were no longer significant upon the inclusion of parents’ ER in the models. Although the present findings differ from previous studies that found a positive relationship between child factors and parents’ mental health and wellbeing (Hastings et al., 2005; Ozturk et al., 2014; Foody et al., 2015; Cohrs and Leslie, 2017; Li et al., 2022) they are consistent with other studies findings that parent-related factors (e.g., increased social support, parent cognition, parents’ internal locus of control) have been largely associated with positive mental health in parents of autistic children (Bishop et al., 2007; Falk et al., 2014; Bitsika and Sharpley, 2016). This difference may be in part due to the distinction between caregiving strain and mental health. Caregiving strain, as measured by the CGSQ-SF, refers to “the demands, responsibilities, difficulties” (Brannan et al., 1997) of caregiving for an autistic child. As such, this measure reflects the amount of daily strain experienced by parents, which may be more related to child-level factors such as adaptive functioning and behavioral concerns. In contrast, parents’ mental health refers to a more global experience of psychological distress, such as the experience of symptoms of anxiety and depression, which may be more related to parent-related factors such as parental cognition. Altogether, our findings underscore the need to consider both child- and parent- level factors in examining contributors to mental health and wellbeing in parents of autistic children.

The ability to regulate emotions also emerged as a significant factor in determining parents’ reports of caregiving strain and mental health. In fact, parents’ ability to regulate emotions was the only factor to predict all the parental outcomes - caregiver strain, depression, anxiety, and wellbeing. Additionally, parents’ ER abilities moderated the impact of children’s RRBs and adaptive functioning challenges on caregiving strain. Although the role of ER has not been specifically studied in parents of autistic children, it has been indirectly addressed in the literature within the related construct of parents’ coping skills (i.e., the behavioral and cognitive efforts employed to reduce distress; Lazarus and Folkman, 1984). From a theoretical perspective, ER and coping strategies both refer to processes aimed at fostering positive emotional states and regulating negative ones (Gross, 2002, 2015). The findings of the current study corroborate previous studies emphasizing the importance of positive coping strategies that address emotion dysregulation, such as cognitive reappraisal or reframing, for reducing stress and psychological distress in parents of autistic children (Hastings et al., 2005; Zablotsky et al., 2013; Shepherd et al., 2018). Conversely, certain coping strategies, such as avoidance or escape behaviors, have been associated with higher levels of stress (Dunn et al., 2001; Hastings et al., 2005). Our findings contribute to the extant literature by examining parents’ overall ability to regulate emotions, as opposed to the utility of specific coping strategies, in relation to the experience of caregiver strain and mental health and wellbeing. Our findings highlight the impact of ER abilities on the experience of caregiver strain and mental health in parents of autistic children and identify parents’ ER abilities as a possible intervention target. The importance of parent ER underscored in the present study also suggests that more environmental supports for parents of autistic children may be critical to providing space and time for parents to regulate complex emotions.

Lastly, a significant difference was seen in the experience of caregiving strain and anxiety reported by mothers and fathers, such that mothers reported higher levels of caregiving strain and anxiety than did fathers. These findings are consistent with previous studies reporting significantly higher levels of caregiver strain and anxiety for mother than fathers (Hastings, 2003; Dabrowska and Pisula, 2010; Foody et al., 2015; Vitale et al., 2022). One possible explanation for this discrepancy is that mothers tend to be more involved in the day-to-day management of family life (Konstantareas and Homaditis, 1992; Benson et al., 2008). In our sample, almost half of mothers identifying as stay-at-home caretakers in comparison to less than 5 percent of fathers. Research has found that greater involvement in managing children’s day-to-day activities is associated with higher levels of caregiver strain (Tehee et al., 2009), which may explain the higher incidence of caregiver strain in mothers. Differences have also been reported in the sources of caregiving strain for parents of autistic children, with mothers’ caregiver strain being predicted by daily living skills (e.g., sleeping, eating) and dysregulation, whereas externalizing behaviors have been found to predict fathers’ caregiver strain (Davis and Carter., 2008). These differences may have contributed to the sex differences seen for caregiving strain and anxiety. In sum, these findings contribute to the extant literature by replicating previous findings in a large sample. Further examination is needed to better understand the child and parent factors that contribute to different experiences of caregiver strain and anxiety in mothers and fathers of autistic children.

### Future directions and limitations

The current study presents findings from a large, national sample of autistic children and their biological parents. These findings are the first to shed light on the importance of ER on the experience of caregiver strain and adverse mental health in parents of autistic children. Despite the considerable strengths of this study, some limitations should be addressed. First, the sample included in this study was majority married, White, with a high SES, which affects the generalizability of the findings. Similarly, parents in this study were in heterosexual relationships, with almost half of mothers identifying as full-time care providers, which affects the generalizability of these findings to other family structures. Additionally, findings are based solely on parent-reports of their own caregiver strain, mental health, and ER, as well as their children’s autism symptomatology, adaptive functioning, and behaviors. Future research should consider other methods of measurement (e.g., direct observation, clinical assessment) when assessing the associations between these constructs. For example, studies examining the association between caregiver strain and children’s RRBs could benefit from the use of observational measures such as the ADOS-2 (Lord et al., 1999), the gold standard for autism diagnosis. Further, future research would benefit from the inclusion of a measure of social communication abilities in autistic children, to further parse the contribution of each core feature of autism. Lastly, while this study begins to examine the ways that ER abilities impact caregiver strain and mental health in parents of autistic children, this area of study is new and further examination is warranted.

## Conclusion

This study is the first to examine how parents’ ER abilities moderate the association between children’s behaviors and parents’ experience of caregiving strain and mental health. Our findings suggest that parents may benefit from supports to improve their ER abilities and environmental supports to provide parents time and space for emotion management, which could improve their ability to cope with day-to-day stressors associated with caregiving for an autistic child.

## Data availability statement

The original contributions presented in this study are included in the article/Supplementary material, further inquiries can be directed to the corresponding author.

## Ethics statement

The studies involving human participants were reviewed and approved by the UCLA IRB Board. The patients/participants provided their written informed consent to participate in this study.

## Author contributions

AD, RF, AS, LV, AW, LA, and AG conceptualized the study. ST and MP conducted the data analysis. AD and RF wrote the manuscript. All authors read and provided feedback on the final manuscript.

## Footnotes

1 Many self-advocates from the autism community (Bury et al., 2020) and current research has indicated both a preference for identify first language (e.g., autistic children) or language describing the individual. Therefore, this language will be utilized throughout the present manuscript.

## References

[B1] Abbeduto L. Seltzer M. M. Shattuck P. Krauss M. W. Orsmond G. Murphy M. M. (2004). Psychological well-being and coping in mothers of youths with autism, down syndrome, orfragile X syndrome. *Am. J. Ment. Retard.* 109 237–254. 10.1352/0895-8017(2004)109<237:PWACIM>2.0.CO;2 15072518

[B2] Achenbach T. M. Rescorla L. A. (2001). *Manual for ASEBA school-age forms & profiles.* Burlington, VT: University of Vermont.

[B3] Anuradha K. (2004). Empowering families with mentally Ill members: A strengths perspective. *Int. J. Adv. Couns.* 26 383–391. 10.1007/s10447-004-0174-x

[B4] Baker J. K. Messinger D. S. Lyons K. K. Grantz C. J. (2010). A pilot study of maternal sensitivity in the context of emergent autism. *J. Autism Dev. Disord.* 40 988–999. 10.1007/s10803-010-0948-4 20130975 PMC2904821

[B5] Barakat L. P. Linney J. A. (1992). Children with physical handicaps and their mothers: The interrelation of social support, maternal adjustment, and child adjustment. *J. Pediatr. Psychol.* 17 725–739. 10.1093/jpepsy/17.6.725 1484335

[B6] Barkley R. A. (2011b). *Barkley Deficits in Executive Functioning Scale (BDEFS for adults).* New York, NY: Guilford Press. 10.1037/t37378-000

[B7] Bayley N. (2006). *Bayley scales of infant and toddler development*. San Antonio, TX: Harcourt Assessment.

[B8] Bendixen R. M. Elder J. H. Donaldson S. Kairalla J. A. Valcante G. Ferdig R. E. (2011). Effects of a father-based in-home intervention on perceived stress and family dynamics in parents of children with autism. *Am. J. Occup. Ther.* 65 679–687. 10.5014/ajot.2011.001271 22214112 PMC3252214

[B9] Benson P. R. Karlof K. L. (2009). Anger, stress proliferation, and depressed mood among parents of children with ASD: A longitudinal replication. *J. Autism Dev. Disord.* 39 350–362. 10.1007/s10803-008-0632-0 18709548

[B10] Benson P. Karlof K. L. Siperstein G. N. (2008). Maternal involvement in the education of young children with autism spectrum disorders. *Autism* 12 47–63. 10.1177/1362361307085269 18178596

[B11] Bishop S. L. Richler J. Cain A. C. Lord C. (2007). Predictors of perceived negative impact in mothers of children with autism spectrum disorder. *Am. J. Ment. Retard.* 112 450–461. 10.1352/0895-8017(2007)112[450:POPNII]2.0.CO;2 17963436

[B12] Bitsika V. Sharpley C. F. (2016). Which aspects of challenging behaviour are associated with anxiety across two age groups of young males with an autism spectrum disorder? *J. Dev. Phys. Disabil.* 28, 685–701. 10.1007/s10882-016-9502-4

[B13] Bodfish J. W. Symons F. J. Lewis M. H. (1999). *The Repetitive Behavior Scales (RBS)*. Research Reports. Chapel Hill, NC: Western Carolina Center.

[B14] Brannan A. M. Athay M. M. de Andrade A. R. V. (2012). Measurement quality of the caregiver strain questionnaire-short form 7 (CGSQ-SF7). *Adm. Policy Ment. Health Ment. Health Serv. Res.* 39 51–59. 10.1007/s10488-012-0412-1 22407562

[B15] Brannan A. M. Heflinger C. A. Bickman L. (1997). The caregiver strain questionnaire: Measuring the impact on the family of living with a child with serious emotional disturbance. *J. Emot. Behav. Disord.* 5 212–222. 10.1177/106342669700500404

[B16] Bristol M. Gallagher J. Schopler E. (1988). Mothers and fathers of young developmentally disabled and nondisabled boys: Adaptation and spousal support. *Dev. Psychol.* 24 441–451. 10.1037/0012-1649.24.3.441

[B17] Bury S. M. Jellett R. Spoor J. R. Hedley D. (2020). “It defines who I am” or “It’s something I have”: What language do [autistic] Australian adults [on the autism spectrum] prefer? *J. Autism Dev. Disord.* 53 677–687. 10.1007/s10803-020-04425-3 32112234

[B18] Clarke P. J. Marshall V. W. Ryff C. D. Wheaton B. (2001). Measuring psychological well-being in the Canadian study of health and aging. *Int. Psychogeriatr.* 13 79–90. 10.1017/S1041610202008013 11892978

[B19] Cludius B. Mennin D. Ehring T. (2020). Emotion regulation as a transdiagnostic process. *Emotion* 20:37. 10.1037/emo0000646 31961175

[B20] Cohrs A. C. Leslie D. L. (2017). Depression in parents of children diagnosed with autism spectrum disorder: A claims-based analysis. *J. Autism Dev. Disord.* 47 1416–1422. 10.1007/s10803-017-3063-y 28214978

[B21] Dabrowska A. Pisula E. (2010). Parenting stress and coping styles in mothers and fathers of pre-school children with autism and Down syndrome. *J. Intell. Disabil. Res.* 54 266–280. 10.1111/j.1365-2788.2010.01258.x 20146741

[B22] Davis N. O. Carter A. S. (2008). Parenting stress in mothers and fathers of toddlers with autism spectrum disorders: Associations with child characteristics. *J. Autism Dev. Disord.* 38 1278–1291. 10.1007/s10803-007-0512-z 18240012

[B23] Dunn M. E. Burbine T. Bowers C. A. Tantleff-Dunn S. (2001). Moderators of stress in parents of children with autism. *Commun. Ment. Health J.* 37 39–52. 10.1023/a:1026592305436 11300666

[B24] Ekas N. V. Pruitt M. M. McKay E. (2016). Hope, social relations, and depressive symptoms in mothers of children with autism spectrum disorder. *Res. Autism Spectr. Disord.* 29 8–18. 10.1016/j.rasd.2016.05.006

[B25] Estes A. Munson J. Dawson G. Koehler E. Zhou X. H. Abbott R. (2009). Parenting stress and psychological functioning among mothers of preschool children with autism and developmental delay. *Autism* 13 375–387. 10.1177/1362361309105658 19535467 PMC2965631

[B26] Estes A. Olson E. Sullivan K. Greenson J. Winter J. Dawson G. (2013). Parenting-related stress and psychological distress in mothers of toddlers with autism spectrum disorders. *Brain Dev.* 35 133–138. 10.1016/j.braindev.2012.10.004 23146332 PMC3552060

[B27] Etkin A. Prater K. E. Hoeft F. Menon V. Schatzberg A. F. (2010). Failure of anterior cingulate activation and connectivity with the amygdala during implicit regulation of emotional processing in generalized anxiety disorder. *Am. J. Psychiatry* 167 545–554. 10.1176/appi.ajp.2009.09070931 20123913 PMC4367202

[B28] Factor R. S. Swain D. M. Scarpa A. (2017). Child autism spectrum disorder traits and parenting stress: The utility of using a physiological measure of parental stress. *J. Autism Dev. Disord.* 48 1081–1091. 10.1007/s10803-017-3397-5 29164443

[B29] Falk N. H. Norris K. Quinn M. G. (2014). The factors predicting stress, anxiety and depression in the parents of children with autism. *J. Autism Dev. Disord.* 44 3185–3203. 25022253 10.1007/s10803-014-2189-4

[B30] Feliciano P. Zhou X. Astrovskaya I. Turner T. N. Wang T. Brueggeman L. (2019). Exome sequencing of 457 autism families recruited online provides evidence for autism risk genes. *NPJ Genomic Med.* 4 1–14. 10.1038/s41525-019-0093-8 31452935 PMC6707204

[B31] Foody C. James J. E. Leader G. (2015). Parenting stress, salivary biomarkers, and ambulatory blood pressure: A comparison between mothers and fathers of children with autism spectrum disorders. *J. Autism Dev. Disord.* 45 1084–1095. 10.1007/s10803-014-2263-y 25287900

[B32] Gabriels R. L. Cuccaro M. L. Hill D. E. Ivers B. J. Goldson E. (2005). Repetitive behaviors in autism: Relationships with associated clinical features. *Res. Dev. Disabil.* 26 169–181. 15590247 10.1016/j.ridd.2004.05.003

[B33] Greenberg J. S. Seltzer M. M. Hong J. Orsmond G. I. (2006). Bidirectional effects of expressed emotion and behavior problems and symptoms in adolescents and adults with autism. *Am. J. Ment. Retard.* 111 229–249. 16792426 10.1352/0895-8017(2006)111[229:BEOEEA]2.0.CO;2

[B34] Gross J. J. (1998). The emerging field of emotion regulation: An integrative review. *Rev. Gen. Psychol.* 2 271–299.

[B35] Gross J. J. (2002). Emotion regulation: Affective, cognitive, and social consequences. *Psychophysiology* 39 281–291. 12212647 10.1017/s0048577201393198

[B36] Gross J. J. (2015). Emotion regulation: Current status and future prospects. *Psychol. Inq.* 26 1–26. 10.1254/fpj.151.21 29321392

[B37] Gulsrud A. C. Jahromi L. B. Kasari C. (2010). The co-regulation of emotions between mothers and their children with autism. *J. Autism Dev. Disord.* 40 227–237. 19714458 10.1007/s10803-009-0861-x PMC2810360

[B38] Haga S. M. Kraft P. Corby E. K. (2009). Emotion regulation: Antecedents and well-being outcomes of cognitive reappraisal and expressive suppression in cross-cultural samples. *J. Happiness Stud.* 10 271–291.

[B39] Harrison P. L. Oakland T. (2015). *ABAS-3.* Torrance, CA: Western Psychological Services.

[B40] Harrop C. McBee M. Boyd B. A. (2016). How are child restricted and repetitive behaviors associated with caregiver stress over time? A parallel process multilevel growth model. *J. Autism Dev. Disord.* 46 1773–1783. 10.1007/s10803-016-2707-7 26801776

[B41] Hartley S. L. Seltzer M. M. Head L. Abbeduto L. (2012). Psychological well-being in fathers of adolescents and young adults with Down Syndrome. Fragile X syndrome, and autism. *Fam. Relat.* 61 327–342. 10.1111/j.1741-3729.2011.00693.x 22611299 PMC3352598

[B42] Hartley S. Papp L. Blumenstock S. Floyd F. Goetz G. (2016). The effect of daily challenges in children with autism on parents’ couple problem-solving interactions. *J. Fam. Psychol.* 30 732–742. 10.1037/fam0000219 27336179 PMC5014690

[B43] Hastings R. P. (2003). Child behaviour problems and partner mental health as correlates of stress in mothers and fathers of children with autism. *J. Intell. Disabil. Res.* 47 231–237. 10.1046/j.1365-2788.2003.00485.x 12787155

[B44] Hastings R. P. Kovshoff H. Brown T. Ward N. J. Espinosa F. D. Remington B. (2005). Coping strategies in mothers and fathers of preschool and school-age children with autism. *Autism* 9 377–391. 10.1177/1362361305056078 16155055

[B45] He B. Wongpakaran T. Wongpakaran N. Wedding D. (2022). Marital satisfaction and perceived family support in families of children with autistic spectrum disorder: Dyadic analysis. *Healthcare* 10:1227. 10.3390/healthcare10071227 PMC9322168 35885754

[B46] Hu X. Han Z. R. Bai L. Gao M. M. (2019). The mediating role of parenting stress in the relations between parental emotion regulation and parenting behaviors in Chinese families of children with autism spectrum disorders: A dyadic analysis. *J. Autism Dev. Disord.* 49 3983–3998. 10.1007/s10803-019-04103-z 31197635 PMC6751273

[B47] Hutchison L. Feder M. Abar B. Winsler A. (2016). Relations between parenting stress, parenting style, and child executive functioning for children with ADHD or autism. *J. Child Fam. Stud.* 25 3644–3656.

[B48] Ingersoll B. Hambrick D. Z. (2011). The relationship between the broader autism phenotype, child severity, and stress and depression in parents of children with autism spectrum disorders. *Res. Autism Spectr. Disord.* 5 337–344. 10.1002/aur.170 21480539

[B49] Karst J. S. Van Hecke A. V. (2012). Parent and family impact of autism spectrum disorders: A review and proposed model for intervention evaluation. *Clin. Child Fam. Psychol. Rev.* 15 247–277. 10.1007/s10567-012-0119-6 22869324

[B50] Keyes C. L. Shmotkin D. Ryff C. D. (2002). Optimizing well-being: The empirical encounter of two traditions. *J. Pers. Soc. Psychol.* 82:1007. 12051575

[B51] Khanjani M. Sohrabi F. Aazami Y. (2018). The effectiveness of resilience and stress management training program on psychological well-being, meaning of life, optimism, and satisfaction of life in female-headed households. *Iran. J. Psychiat. Nurs.* 6 1–11.

[B52] Konstantareas M. M. Homaditis S. (1992). Mothers’ and fathers’ self-report of involvement with autistic, mentally delayed, and normal children. *J. Marriage Fam.* 54 153–164. 10.2307/353283

[B53] Kroenke K. Spitzer R. L. (2002). The PHQ-9: A new depression diagnostic and severity measure. *Psychiatr. Ann.* 32 509–515. 10.3928/0048-5713-20020901-06

[B54] Lazarus R. S. Folkman S. (1984). *Stress, appraisal, and coping.* Berlin: Springer Publishing Company.

[B55] Lecavalier L. Leone S. Wiltz J. (2006). The impact of behaviour problems on caregiver stress in young people with autism spectrum disorders. *J. Intell. Disabil. Res.* 50 172–183. 10.1111/j.1365-2788.2005.00732.x 16430729

[B56] Li F. Tang Y. Li F. Fang S. Liu X. Tao M. (2022). Psychological distress in parents of children with autism spectrum disorder: A cross-sectional study based on 683 mother-father dyads. *J. Pediatr. Nurs.* 65 e49–e55. 10.1016/j.pedn.2022.02.006 35249769

[B57] Lord C. Rutter M. DiLavore P. C. Risi S. (1999). *Autism diagnostic observation schedule-WPS (WPS edition)*. Los Angeles, CA: Western Psychological Services.

[B58] Luthar S. S. Cicchetti D. Becker B. (2000). The construct of resilience: A critical evaluation and guidelines for future work. *Child Dev.* 71 543–562. 10.1111/1467-8624.00164 10953923 PMC1885202

[B59] Lyons A. M. Leon S. C. Roecker Phelps C. E. Dunleavy A. M. (2010). The impact of child symptom severity on stress among parents of children with ASD: The moderating role of coping styles. *J. Child Fam. Stud.* 19 516–524.

[B60] Mandal S. P. Arya Y. K. Pandey R. (2011). Mindfulness, emotion regulation and subjective wellbeing: An overview of pathways to positive mental health. *Indian J. Soc. Sci. Res.* 8 159–167.

[B61] Mennin D. S. Holaway R. M. Fresco D. M. Moore M. T. Heimberg R. G. (2007). Delineating components of emotion and its dysregulation in anxiety and mood psychopathology. *Behav. Ther.* 38 284–302. 10.1016/j.beth.2006.09.001 17697853

[B62] Mercier C. Mottron L. Belleville S. (2000). A psychosocial study on restricted interests in high functioning persons with pervasive developmental disorders. *Autism* 4 406–425.

[B63] Moes D. Koegel R. L. Schreibman L. Loos L. M. (1992). Stress profiles for mothers and fathers of children with autism. *Psychol. Rep.* 71(3_Suppl) 1272–1274. 1480714 10.2466/pr0.1992.71.3f.1272

[B64] Nikmat A. W. Ahmad M. Oon N. L. Razali S. (2008). Stress and psychological wellbeing among parents of children with autism spectrum disorder. *ASEAN J. Psychiatry* 9 65–72.

[B65] Ozturk Y. Riccadonna S. Venuti P. (2014). Parenting dimensions in mothers and fathers of children with Autism Spectrum Disorders. *Res. Autism Spectr. Disord.* 8 1295–1306.

[B66] Pepperell T. A. Paynter J. Gilmore L. (2018). Social support and coping strategies of parents raising a child with autism spectrum disorder. *Early Child Dev. Care* 188 1392–1404.

[B67] Porter N. Loveland K. A. Saroukhani S. Posey Y. Morimoto K. Rahbar M. H. (2022). Severity of child autistic symptoms and parenting stress in mothers of children with autism spectrum disorder in Japan and USA: Cross-cultural differences. *Autism Res. Treat.* 2022:7089053. 10.1155/2022/7089053 35864923 PMC9296302

[B68] Postorino V. Gillespie S. Lecavalier L. Smith T. Johnson C. Swiezy N. (2019). Clinical correlates of parenting stress in children with autism spectrum disorder and serious behavioral problems. *J. Child Fam. Stud.* 28 2069–2077.

[B69] Preuss H. Capito K. van Eickels R. L. Zemp M. Kolar D. R. (2021). Cognitive reappraisal and self-compassion as emotion regulation strategies for parents during COVID-19: An online randomized controlled trial. *Intern. Intervent.* 24:100388. 10.1016/j.invent.2021.100388 33912402 PMC8063732

[B70] Rao P. A. Beidel D. C. (2009). The impact of children with high-functioning autism on parental stress, sibling adjustment, and family functioning. *Behav. Modif.* 33 437–451. 10.1177/0145445509336427 19436073

[B71] Rezendes D. L. Scarpa A. (2011). Associations between parental anxiety/depression and child behavior problems related to autism spectrum disorders: The roles of parenting stress and parenting self-efficacy. *Autism Res. Treat.* 2011:395190. 10.1155/2011/395190 22937246 PMC3420762

[B72] Russell B. S. Hutchison M. Park C. L. Fendrich M. Finkelstein-Fox L. (2022). Short-term impacts of COVID-19 on family caregivers: Emotion regulation, coping, and mental health. *J. Clin. Psychol.* 78 357–374. 10.1002/jclp.23228 34331773 PMC8427037

[B73] Ryff C. D. Keyes C. L. M. (1995). The structure of psychological well-being revisited. *J. Pers. Soc. Psychol.* 69:719. 10.1037/0022-3514.69.4.719 7473027

[B74] Sagone E. De Caroli M. E. (2014). Relationships between psychological well-being and resilience in middle and late adolescents. *Proc. Soc. Behav. Sci.* 141 881–887. 10.1016/j.sbspro.2014.05.154

[B75] Seligman M. Darling R. B. (2009). *Ordinary families, special children: A systems approach to childhood disability.* New York, NY: Guilford Publications.

[B76] Sharpley C. F. Bitsika V. Efremidis B. (1997). Influence of gender, parental health, and perceived expertise of assistance upon stress, anxiety, and depression among parents of children with autism. *J. Intell. Dev. Disabil.* 22 19–28. 10.1080/13668259700033261

[B77] Shepherd D. Landon J. Taylor S. Goedeke S. (2018). Coping and care-related stress in parents of a child with autism spectrum disorder. *Anxiety Stress Coping* 31 277–290. 10.1080/10615806.2018.1442614 29463108

[B78] Slattery É McMahon J. Gallagher S. (2017). Optimism and benefit finding in parents of children with developmental disabilities: The role of positive reappraisal and social support. *Res. Dev. Disabil.* 65 12–22. 10.1016/j.ridd.2017.04.006 28432893

[B79] Smith L. E. Greenberg J. S. Mailick M. R. (2012). Adults with autism: Outcomes, family effects, and the multi-family group psychoeducation model. *Curr. Psychiatry Rep.* 14 732–738. 10.1007/s11920-012-0328-1 23015048 PMC3492520

[B80] Smith L. E. Greenberg J. S. Seltzer M. M. Hong J. (2008). Symptoms and behavior problems of adolescents and adults with autism: Effects of mother-child relationship quality, warmth, and praise. *Am. J. Ment. Retard.* 113, 387–402. 10.1352/2008.113:387-402 18702558 PMC2826841

[B81] Sparrow S. Cicchetti D. Saulnier C. (2016). *Vineland adaptive behavior scales*, 3rd Edn. Thousand Oaks, CA: SAGE.

[B82] Spitzer R. L. Kroenke K. Williams J. B. Löwe B. (2006). A brief measure for assessing generalized anxiety disorder: The GAD-7. *Arch. Intern. Med.* 166 1092–1097. 10.1001/archinte.166.10.1092 16717171

[B83] Springer K. W. Hauser R. M. (2006). An assessment of the construct validity of Ryff’s scales of psychological well-being: Method, mode, and measurement effects. *Soc. Sci. Res.* 35 1080–1102. 10.1016/j.ssresearch.2005.07.004

[B84] Tehee E. Honan R. Hevey D. (2009). Factors contributing to stress in parents of individuals with autistic spectrum disorders. *J. Appl. Res. Intell. Disabil.* 22 34–42. 10.1111/j.1468-3148.2008.00437.x

[B85] Vitale S. R. Schneider H. Gardner L. Alessandri M. Marker C. (2022). Challenging behavior and parental depression: The effects of everyday stressors and benefit finding for parents of children with autism spectrum disorder. *J. Autism Dev. Disord.* 1–13. 10.1007/s10803-022-05627-7 35749002

[B86] Weiss J. A. Cappadocia M. C. MacMullin J. A. Viecili M. Lunsky Y. (2012). The impact of child problem behaviors of children with ASD on parent mental health: The mediating role of acceptance and empowerment. *Autism* 16 261–274. 10.1177/1362361311422708 22297202

[B87] Wilgosh L. Scorgie K. (2006). Theoretical model for conceptualizing cross-cultural applications and intervention strategies for parents of children with disabilities. *J. Policy Pract. Intell. Disabil.* 3 211–218.

[B88] Zablotsky B. Bradshaw C. P. Stuart E. A. (2013). The association between mental health, stress, and coping supports in mothers of children with autism spectrum disorders. *J. Autism Dev. Disord.* 43 1380–1393. 10.1007/s10803-012-1693-7 23100053

