# Supplementary material for: Examining indicators of psychosocial risk and resilience in parents of autistic children
Source: Front Behav Neurosci. 2023 May 15;17:1102516. doi: 10.3389/fnbeh.2023.1102516 (PMC10226532; doi:10.3389/fnbeh.2023.1102516)
Supplement: Supplementary file 1 [file Table_1.docx]

|  | CGSQ | | | GAD-7 | | | PHQ-9 | | | WBS | | |
| --- | --- | --- | --- | --- | --- | --- | --- | --- | --- | --- | --- | --- |
|  | *Estimates* | *CI* | *P* | *Estimates* | *CI* | *P* | *Estimates* | *CI* | *P* | *Estimates* | *CI* | *P* |
| Intercept | 26.09  *** | 24.98 – 27.19 | <.001 | 6.66  *** | 5.98 – 7.34 | <.001 | 6.78  *** | 6.04 – 7.53 | <.001 | 5.02  *** | 4.91 – 5.13 | <.001 |
| VABS | -2.39  *** | -3.52 –  -1.25 | <.001 | 0.07 | -0.63 – 0.78 | .839 | 0.51 | -0.25 – 1.27 | .191 | 0.01 | -0.10 – 0.12 | .919 |
| CBCL | -1.14 | -2.25 –  -0.04 | .043 | -0.08 | -0.78 – 0.61 | .814 | -0.09 | -0.85 – 0.67 | .810 | -0.06 | -0.16 – 0.05 | .298 |
| RBS-R | 1.97  ** | 0.83 – 3.11 | .001 | 0.66 | -0.04 – 1.36 | .065 | 0.75 | -0.01 – 1.51 | .054 | -0.08 | -0.19 – 0.03 | .153 |
| Parent | 2.04  ** | 0.90 -3.19 | <.001 | 1.65  ** | 0.75 – 2.56 | <.001 | 1.00 | 0.08 – 1.93 | .033 | 0.05 | -0.08 – 0.18 | .453 |
| VABS*  Parent | -0.36 | -1.53 – 0.82 | .553 | -0.12 | -1.05 – 0.81 | .799 | -0.72 | -1.67 – 0.22 | .135 | 0.02 | -0.12 – 0.16 | .770 |
| CBCL*  Parent | 0.89 | -0.25 – 2.03 | .125 | 0.21 | -0.70 – 1.11 | .657 | -0.04 | -0.97 – 0.89 | .927 | 0.03 | -0.10 – 0.16 | .649 |
| RBS-R*  Parent | 0.29 | -0.88 – 1.47 | .622 | 0.51 | -0.42 – 1.44 | .281 | 0.44 | -0.50 – 1.38 | .361 | -0.09 | -0.23 – 0.04 | .178 |

**Supplement**

*Table 1: Results of linear mixed effect models evaluating the effect of child characteristics on the four outcomes of interest, including all two-way interactions with parent. Bonferroni-corrected significance levels were used to control the Type I error rate across the four outcome models.*

*Note: * p<0.0125; ** p< 0.0025; *** p < 0.00025*

*Table 2: Results of linear mixed effect models evaluating the moderating effect of Emotion Regulation on the association between child characteristics on the four outcomes of interest, including all two- and three-way interactions with parent and emotion. Bonferroni-corrected significance levels were used to control the Type I error rate across the four outcome models.*

|  | CGSQ | | | GAD-7 | | | PHQ-9 | | | WBS | | |
| --- | --- | --- | --- | --- | --- | --- | --- | --- | --- | --- | --- | --- |
|  | *Estimates* | *CI* | *P* | *Estimates* | *CI* | *P* | *Estimates* | *CI* | *P* | *Estimates* | *CI* | *P* |
| Intercept | 25.75  *** | 24.67-26.82 | <.001 | 6.67  *** | 6.11-7.24 | <.001 | 6.74  *** | 6.11-7.38 | <.001 | 5.02  *** | 4.93-5.11 | <.001 |
| VABS | -2.47  *** | -3.61- -1.33 | <.001 | -0.65 | -1.26- -0.05 | .033 | -0.14 | -0.82- 0.53 | .672 | 0.08 | -0.02- 0.18 | .118 |
| CBCL | -1.03 | -2.10- 0.04 | .059 | 0.12 | -0.45- 0.68 | .684 | 0.09 | -0.55- 0.72 | .790 | -0.07 | -0.17- 0.02 | .120 |
| RBS-R | 1.63* | 0.52- 2.73 | .004 | 0.18 | -0.39- 0.76 | .529 | 0.25 | -0.39- 0.89 | .441 | -0.01 | -0.11- 0.08 | .765 |
| Parent | 2.44  *** | 1.36- 3.51 | <.001 | 1.66  *** | 0.92- 2.41 | <.001 | 1.01  * | 0.23- 1.79 | .012 | 0.04 | -0.08- 0.16 | .479 |
| Emotion | 2.16  *** | 1.18- 3.14 | <.001 | 3.52  *** | 2.93- 4.12 | <.001 | 3.71  *** | 3.06- 4.36 | <.001 | -0.48  *** | -0.58- -0.38 | <.001 |
| VABS*  Parent | -0.28 | -1.41- 0.85 | .631 | 0.60 | -0.18- 1.38 | .133 | -0.07 | -0.89- 0.75 | .861 | -0.05 | -0.18- 0.08 | .443 |
| VABS*  ER | 1.22  * | 0.27- 2.16 | .011 | -0.57 | -1.14- 0.00 | .051 | -0.16 | -0.78- 0.47 | .620 | 0.02 | -0.08- 0.12 | .703 |
| CBCL*  Parent | 0.72 | -0.33- 1.78 | .178 | 0.03 | -0.71- 0.76 | .943 | -0.25 | -1.03- 0.52 | .524 | 0.05 | -0.07- 0.17 | .398 |
| CBCL*  ER | -0.92 | -1.87- 0.02 | .055 | 0.01 | -0.56- 0.58 | .969 | 0.19 | -0.43- 0.81 | .553 | -0.04 | -0.14- 0.05 | .396 |
| RBS-R*  Parent | 0.28 | -0.82- 1.39 | .613 | 0.54 | -0.23- 1.31 | .167 | 0.50 | -0.30- 1.30 | .224 | -0.09 | -0.21- 0.04 | .163 |
| RBS-R*  ER | 1.32  ** | 0.47- 2.17 | .002 | 0.11 | -0.40- 0.62 | .671 | 0.18 | -0.38- 0.73 | .536 | -0.03 | -0.11- 0.06 | .527 |
| Parent*  ER | -0.09 | -1.44- 1.25 | .891 | -0.51 | -1.34- 0.33 | .233 | -0.92 | -1.83- -0.02 | .045 | 0.08 | -0.06- 0.22 | .253 |
| VABS*  Parent*  ER | -0.38 | -1.72- 0.96 | .582 | 0.85 | 0.02- 1.68 | .044 | 0.59 | -0.31- 1.49 | .199 | -0.06 | -0.20- 0.08 | .434 |
| CBCL*  Parent*  ER | 1.26 | 0.06- 2.47 | .040 | -0.18 | -0.94- 0.59 | .651 | -0.37 | -1.19- 0.46 | .381 | 0.03 | -0.10- 0.15 | .662 |
| RBS-R*  Parent*  ER | -1.49 | -2.70- -0.28 | .016 | 0.20 | -0.55- 0.94 | .605 | 0.19 | -0.61- 1.00 | .641 | 0.04 | -0.08- 0.17 | .481 |

*Note: * p<0.0125; ** p< 0.0025; *** p < 0.00025*
